# Prognostic impact of estimated remnant-like particle cholesterol in patients with differing glycometabolic status: an observational cohort study from China

**DOI:** 10.1186/s12944-020-01355-y

**Published:** 2020-07-31

**Authors:** Qi Zhao, Ting-Yu Zhang, Yu-Jing Cheng, Yue Ma, Ying-Kai Xu, Jia-Qi Yang, Yu-Jie Zhou

**Affiliations:** 1 Department of Cardiology, Beijing Anzhen Hospital, Capital Medical University, Beijing, 100029 China; 2 Beijing Institute of Heart Lung and Blood Vessel Disease, Beijing Key Laboratory of Precision Medicine of Coronary Atherosclerotic Disease, Clinical center for coronary heart disease, Capital Medical University, Beijing, 100029 China; 3 Research Center for Coronary Heart Disease, Fuwai Hospital, National Center for Cardiovascular Diseases, Chinese Academy of Medical Sciences and Peking Union Medical College, Beijing, 100037 China

**Keywords:** Remnant-like particle cholesterol, Non-ST-segment elevation acute coronary syndrome, Percutaneous coronary intervention

## Abstract

**Background:**

It is uncertain whether estimated remnant-like particle cholesterol (RLP-C) could predict residual risk in patients with different glycometabolic status. This study aimed to evaluate the relationship between estimated RLP-C and adverse prognosis in patients with non-ST-segment elevation acute coronary syndrome (NSTE-ACS) treated with percutaneous coronary intervention (PCI) and to identify the potential impact of glycometabolism on the predictive value of estimated RLP-C.

**Methods:**

The study assessed 2419 participants with NSTE-ACS undergoing PCI at Beijing Anzhen Hospital from January to December 2015. Estimated RLP-C was calculated as follows: total cholesterol (TC) minus low-density lipoprotein cholesterol (LDL-C) and high-density lipoprotein cholesterol (HDL-C). The adverse events included all-cause death, non-fatal myocardial infarction (MI), and ischemia-driven revascularization.

**Results:**

Estimated RLP-C was prominently associated with adverse prognosis in the total population [hazard ratio (HR) 1.291 per 1-SD increase, 95% confidence interval (CI) 1.119–1.490, *P* <  0.001], independent of confounding risk factors. However, subgroup analysis showed that increasing estimated RLP-C was related to a higher risk of adverse events in the diabetic population only [HR 1.385 per 1-SD increase, 95% CI 1.183–1.620, *P* <  0.001]. Estimated RLP-C failed to be a significant determinant of adverse prognosis in non-diabetic and pre-diabetic subgroups. The addition of estimated RLP-C to a baseline model including traditional risk factors enhanced the predictive performance both in total and diabetic populations.

**Conclusions:**

High estimated RLP-C level is a significant predictor for recurrent adverse events in patients with diabetes and NSTE-ACS treated with PCI.

## Background

As the most serious manifestation of atherosclerotic cardiovascular disease (ASCVD), acute coronary syndrome (ACS) leads to a consistently higher risk of recurrence of cardiovascular outcomes, despite wide application of evidence-based secondary prevention strategies [1, 2]. Low-density lipoprotein cholesterol (LDL-C) has been extensively recognized as the significant risk factor for ASCVD, reduction of which is an effective therapy to reduce cardiovascular risks [3]. Despite regulating LDL-C with guideline-recommended therapies, patients with ACS remain at a higher risk of recurrent cardiovascular outcomes [4–7], which indicates that there are factors other than LDL-C that determine risk.

Studies have reported that the residual risk can be partly ascribed to an increased level of remnant lipoproteins [2, 4, 8, 9]. Remnant lipoproteins are lipoproteins that are rich in triglycerides (TGs), components of which include chylomicron remnant, very-low-density lipoprotein (VLDL), and intermediate-density lipoprotein (IDL) [10]. The cholesterol content of remnant lipoproteins is defined as remnant-like particle cholesterol (RLP-C). Nowadays, the pattern of targeting LDL-C alone has changed, with recent guidelines highlighting the importance of non-high-density lipoprotein cholesterol (non-HDL-C), which includes RLP-C, on the pathogenesis of atherosclerosis and thus its availability as an additional therapeutic target [11]. As a component of non-HDL-C, it is of great significance to further clarify the effect of RLP-C on coronary atherosclerosis.

The prognostic significance of RLP-C on adverse prognosis in the specific cohort diagnosed with non-ST-segment elevation acute coronary syndrome (NSTE-ACS) and treated with percutaneous coronary intervention (PCI) were not explicitly investigated. Results from former studies revealed that the prognostic impact of RLP-C seems to be more prominent in patients with metabolic syndrome or type 2 diabetes [12–16]. It is worth exploring whether the prognostic value of RLP-C varies among populations with different glycometabolic status. The current study is the first to investigate the relationship between estimated RLP-C and recurrent adverse events, and confirm the potential impact of glycometabolic status on the predictive value of estimated RLP-C in participants with NSTE-ACS undergoing PCI.

## Methods

### Study population

This study retrospectively screened patients diagnosed with NSTE-ACS who received PCI treatment in Beijing Anzhen Hospital (Beijing, China) from January to December 2015. The definition of NSTE-ACS was in accordance with corresponding guidelines [17], including non-ST segment elevation myocardial infarction (NSTEMI) and unstable angina pectoris (UA). The exclusion criteria included: (1) missing clinical, laboratory, and/or angiographic data; (2) history of cardiogenic shock, chronic inflammatory disease, or neoplasm; (3) evidence of active infection; (4) severe renal dysfunction with estimated glomerular filtration rate (eGFR) lower than 30 mL/(min*1.73 m^2^), severe hepatic disease, and other serious diseases; (5) death in hospital, complications or procedure failure of PCI. Based on the enrollment criteria, 2419 participants were finally included in the study.

The study population was divided into three subgroups according to the glycometabolic status. Patients with a definite prior diagnosis of diabetes and glycosylated hemoglobin A1c (HbA1c) ≥ 6.5% on admission were defined as the diabetic population. Patients with HbA1c level < 5.7 and 5.7% ~ 6.4% were considered as non-diabetic and pre-diabetic population, respectively, as previous guidelines mentioned [18].

### Data collection

The enrolled patients’ demographic characteristics, clinical information, laboratory investigations, and coronary procedural results were retrieved and collected by using the medical information recording system from Beijing Anzhen Hospital.

All laboratory parameters were analyzed in the central laboratory of the hospital by using the first fasting venous blood samples before the baseline PCI. High-sensitivity C-reactive protein (hs-CRP), creatinine, uric acid, fasting blood glucose (FBG), and HbA1c were measured by standard methods. Concentrations of TGs, total cholesterol (TC), and high-density lipoprotein cholesterol (HDL-C) were quantified by standard enzymatic methods. LDL-C was determined by the homogeneous direct method. Estimated RLP-C was calculated by subtracting LDL-C and HDL-C from TC, which was recommended by relevant dyslipidemia guidelines [19, 20]. The eGFR was computed by MDRD equation as previously proposed [21]. Two-dimensional Simpson’s method was applied to evaluate the left ventricular ejection fraction (LVEF).

Coronary angiographic data were analyzed and evaluated by visual measurements, and the results were documented and verified by at least two experienced cardiologists. The multi-vessel disease was defined as more than two main epicardial coronary arteries with stenosis ≥50%. The lesion with complete cessation of blood flow [thrombolysis in myocardial infarction (TIMI) flow grade 0] and duration ≥3 months was considered as a chronic total occlusion. An independent lesion with a length ≥ 20 mm was defined as a diffuse lesion, and the lesion implicating the origin of an important side branch was defined as a bifurcation lesion. Coronary procedures were carried out based on the relevant guideline of China [22], and specific procedure strategies were selected by practiced cardiologists.

### Follow-up

After coronary procedures had been performed, all participants received routine follow-up at 3, 6, and 12 months and then annually until 36 months by means of phone interviews with the patient and/or family members. Corresponding medical records were referred to verify the authenticity in case that ambiguous information was obtained. Adverse outcomes including all-cause death, non-fatal myocardial infarction (MI), and ischemia-driven revascularization were recorded as composite or separate adverse event to implement the current analysis. If participants experienced more than one adverse event during the 36-month follow-up, only the first instance of the event was selected to proceed the current study.

### Statistical analysis

Continuous variables were displayed as mean ± SD or median (25th and 75th percentiles), and compared by t-test or Mann-Whitney U test, as appropriate. Nominal variables were expressed as absolute quantities and proportions, and compared by χ2 test or Fisher’s exact test when appropriate. The correlations between estimated RLP-C and other variables were assessed by Pearson or Spearman’s rank correlation test as applicable. The incidence of events in groups with lower and higher median of estimated RLP-C was described by Kaplan-Meier curves, and the difference between groups was compared by log-rank test. The simple Cox analyses were primarily conducted to confirm the significant predictors of adverse events. The variables with statistical significance (*P* <  0.05) in simple Cox analysis were analyzed with multiple Cox analysis to investigate the independent determinants of adverse events. The results of Cox analysis were interpreted using hazard ratio (HR) and 95% confidence intervals (CI). The HR was examined by 1-SD change in continuous variables except for age, heart rate, systolic blood pressure (SBP), and number of stents. C-statistics that consisted of receiver-operating characteristic (ROC) analysis was applied to estimate the additional discriminative ability of estimated RLP-C for predicting worse outcomes on the basis of the baseline model including traditional risk factors. Differences between the area under the ROC curve (AUC) of various models were compared by DeLong’s test. Moreover, the incremental reclassification and discrimination ability of estimated RLP-C beyond the baseline model for predicting adverse events was further determined by category-free net reclassification improvement (NRI) and integrated discrimination improvement (IDI). The population was divided into three subgroups according to glycometabolic status: diabetic, pre-diabetic, and non-diabetic groups. Similar statistical analyses were performed for each subgroup. Statistical analyses were performed using SPSS (version 23.0; IBM, IL, USA), MedCalc Statistical Software (version 19.1; Ostend, Belgium), and the R Project (version 3.5.1). A *P* value of 0.05 was applied to assess statistical significance.

## Results

### Baseline characteristics

The final enrolled 2419 participants (age 60.08 ± 8.97; 71.8% male) were divided into with-event and without-event group, baseline characteristics of which were summarized in Table 1. The level of estimated RLP-C in participants with an adverse event was prominently higher than those without (0.90 ± 0.61 vs. 0.65 ± 0.35, *P* <  0.001). Patients with an adverse event were observed to be older and had higher body mass index (BMI), heart rate, and SBP. The prevalence of prior MI, PCI, coronary artery bypass grafting (CABG), stroke, and diabetes were higher in the group with an event. With regard to laboratory parameters, participants that developed adverse events displayed higher TGs, TC, hs-CRP, creatinine, HbA1c, and FBG, but lower HDL-C, eGFR, and LVEF. In terms of the angiographic information, more complex coronary artery lesions were exhibited and more stents were implanted in participants with an adverse event. In addition, more participants were diagnosed with NSTEMI, and more angiotensin-converting enzyme inhibitors (ACEI), oral hypoglycemic agents, and insulin were prescribed in patients with an adverse event.

**Table 1:** Baseline clinical characteristics of the study population

	Total population, *n* = 2419	Without event, *n* = 1965	With event, *n* = 454	*P*
Age, years	60.08 ± 8.97	59.60 ± 8.72	62.16 ± 9.70	**< 0.001**
Male, n (%)	1737 (71.8)	1422 (72.4)	315 (69.4)	0.203
BMI, kg/m^2^	26.21 ± 3.45	26.13 ± 3.40	26.55 ± 3.61	**0.019**
Heart rate, bpm	69.77 ± 10.15	69.44 ± 10.00	71.17 ± 10.69	**0.002**
SBP, mmHg	130.30 ± 16.52	129.80 ± 15.99	132.44 ± 18.50	**0.005**
DBP, mmHg	77.05 ± 9.90	77.00 ± 9.68	77.25 ± 10.80	0.661
Smoking, n (%)	1381 (57.1)	1127 (57.4)	254 (55.9)	0.585
Drinking, n (%)	562 (23.2)	468 (23.8)	94 (20.7)	0.157
Family history of CAD, n (%)	254 (10.5)	203 (10.3)	51 (11.2)	0.572
Medical history, n (%)				
Hypertension	1511 (62.5)	1210 (61.6)	301 (66.3)	0.061
Prior MI	527 (21.8)	348 (17.7)	179 (39.4)	**< 0.001**
Prior PCI	414 (17.1)	280 (14.2)	134 (29.5)	**< 0.001**
Prior CABG	55 (2.3)	23 (1.2)	32 (7.0)	**< 0.001**
Prior stroke	281 (11.6)	204 (10.4)	77 (17.0)	**< 0.001**
Prior PAD	84 (3.5)	63 (3.2)	21 (4.6)	0.137
Glycometabolic status				
Non-diabetes	926 (38.3)	829 (42.2)	97 (21.4)	**< 0.001**
Pre-diabetes	645 (26.7)	531 (27.0)	114 (25.1)	0.406
Diabetes	848 (35.1)	605 (30.8)	243 (53.5)	**< 0.001**
Laboratory results				
TGs, mmol/L	1.84 ± 1.32	1.69 ± 1.05	2.47 ± 2.00	**< 0.001**
TC, mmol/L	4.17 ± 1.06	4.14 ± 1.05	4.33 ± 1.07	**0.001**
LDL-C, mmol/L	2.50 ± 0.88	2.50 ± 0.89	2.50 ± 0.85	0.962
HDL-C, mmol/L	0.98 ± 0.23	0.99 ± 0.24	0.92 ± 0.21	**< 0.001**
Estimated RLP-C, mmol/L	0.69 ± 0.42	0.65 ± 0.35	0.90 ± 0.61	**< 0.001**
hs-CRP, mg/L	1.29 (0.58, 3.31)	1.22 (0.53, 3.06)	1.87 (0.77, 4.29)	**< 0.001**
Creatinine, μmol/L	76.00 ± 16.95	75.68 ± 16.49	77.42 ± 18.76	**0.048**
eGFR, ml/(min*1.73m^2^)	93.49 ± 20.36	94.09 ± 20.11	90.91 ± 21.22	**0.003**
Uric acid, μmol/L	346.22 ± 82.64	346.45 ± 81.45	345.21 ± 87.69	0.774
FBG, mmol/L	6.20 ± 1.94	6.01 ± 1.71	7.03 ± 2.57	**< 0.001**
HbA1c, %	5.90 (5.50, 6.60)	5.80 (5.50, 6.40)	6.40 (5.80, 8.00)	**< 0.001**
LVEF, %	65.00 (60.00, 68.00)	65.00 (61.00, 69.00)	63.00 (57.00, 67.00)	**< 0.001**
Initial diagnosis, n (%)				**0.001**
UA	2018 (83.4)	1662 (84.6)	356 (78.4)	
NSTEMI	401 (16.6)	303 (15.4)	98 (21.6)	
Medical treatment, n (%)				
ACEI	734 (30.3)	577 (29.4)	157 (34.6)	**0.029**
ARB	948 (39.2)	753 (38.3)	195 (43.0)	0.068
Aspirin	2417 (99.9)	1963 (99.9)	454 (100.0)	0.496
Clopidogrel	2415 (99.8)	1963 (99.9)	452 (99.6)	0.109
β-Blocker	2199 (90.9)	1780 (90.6)	419 (92.3)	0.255
Statins	2366 (97.8)	1922 (97.8)	444 (97.8)	0.985
Oral hypoglycemic agents	437 (18.1)	314 (16.0)	123 (27.1)	**< 0.001**
Insulin	232 (9.6)	154 (7.8)	78 (17.2)	**< 0.001**
Angiographic data, n (%)				
Left main disease	110 (4.5)	64 (3.3)	46 (10.1)	**< 0.001**
Multi-vessel disease	1631 (67.4)	1225 (62.3)	406 (89.4)	**< 0.001**
Chronic total occlusion	345 (14.3)	202 (10.3)	143 (31.5)	**< 0.001**
Diffuse lesion	605 (25.0)	431 (21.9)	174 (38.3)	**< 0.001**
Bifurcation lesion	492 (20.3)	368 (18.7)	124 (27.3)	**< 0.001**
Number of stents	1.96 ± 1.29	1.87 ± 1.14	2.33 ± 1.76	**< 0.001**

Bold values indicate statistically significant associations

*BMI* Body mass index, *SBP* Systolic blood pressure, *DBP* Diastolic blood pressure, *CAD* Coronary artery disease, *MI* Myocardial infarction, *PCI* Percutaneous coronary intervention, *CABG* Coronary artery bypass grafting, *PAD* Peripheral arterial disease, *TGs* Triglycerides, *TC* Total cholesterol, *LDL-C* Low-density lipoprotein cholesterol, *HDL-C* High-density lipoprotein cholesterol, *RLP-C* Remnant-like particle cholesterol, *hs-CRP* High-sensitivity C-reactive protein, *eGFR* Estimated glomerular filtration rate, *FBG* Fasting blood glucose, *HbA1c* Glycosylated hemoglobin A1c, *LVEF* Left ventricular ejection fraction, *UA* Unstable angina, *NSTEMI* Non-ST-segment elevation myocardial infarction, *ACEI* Angiotensin-converting enzyme inhibitor, *ARB* Angiotensin receptor blocker

**Fig. 1:**
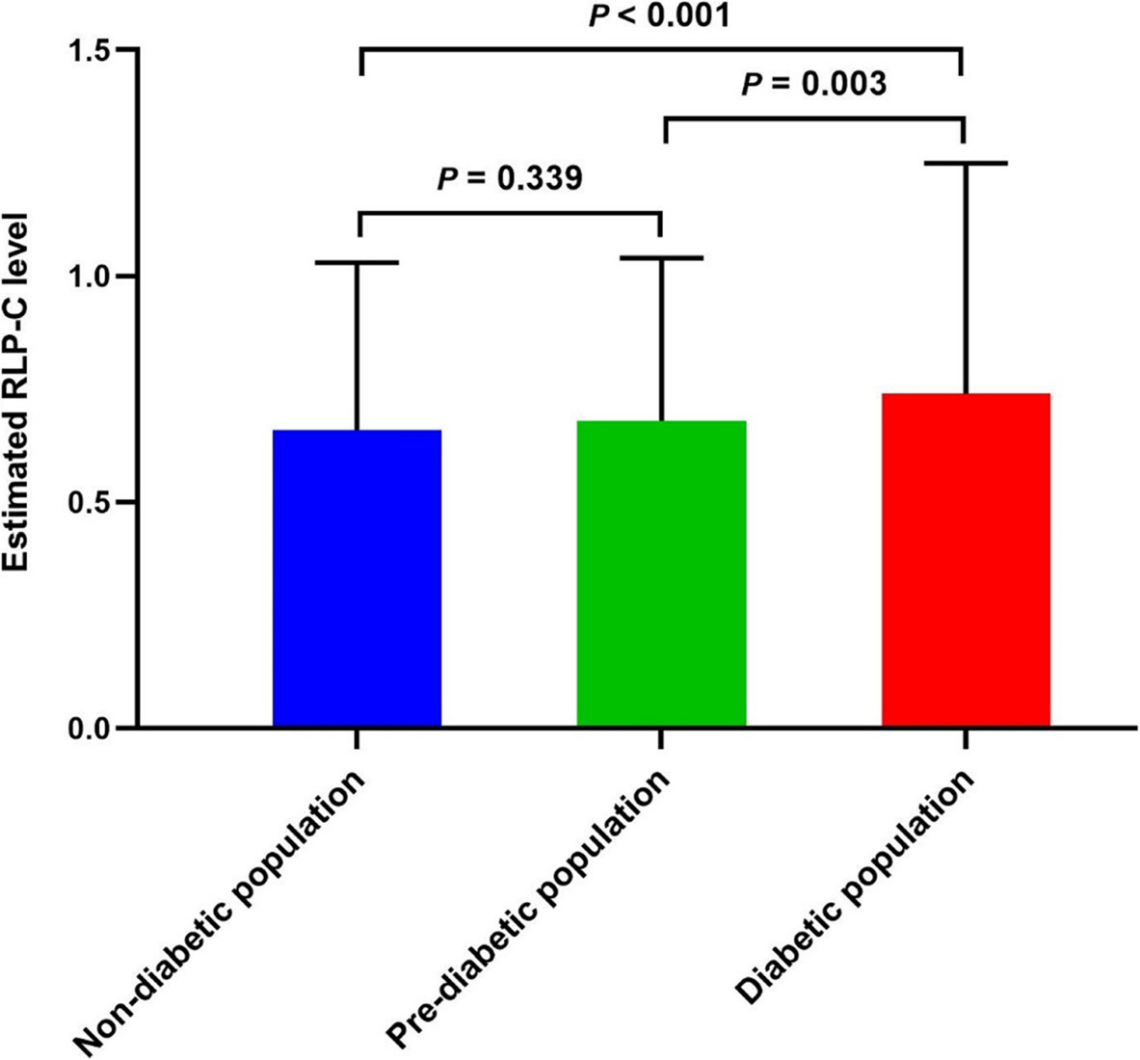
Estimated RLP-C levels in different glycometabolic status. RLP-C, remnant-like particle cholesterol

Estimated RLP-C was higher in participants with diabetes than pre-diabetes (0.74 ± 0.51 vs 0.68 ± 0.36, *P* = 0.003) and non-diabetes (0.74 ± 0.51 vs 0.66 ± 0.37, *P* <  0.001). However, the difference between pre-diabetic and non-diabetic populations was not conspicuous (0.68 ± 0.36 vs 0.66 ± 0.37, *P* = 0.339) (Fig. 1). Estimated RLP-C was positively correlated to TGs (*r* = 0.853, *P* <  0.001), TC (*r* = 0.455, *P* <  0.001), and LDL-C (*r* = 0.112, *P* <  0.001), while negatively correlated to HDL-C (*r* = − 0.173, *P* <  0.001).

### Predictive value of estimated RLP-C in total population

**Fig. 2:**
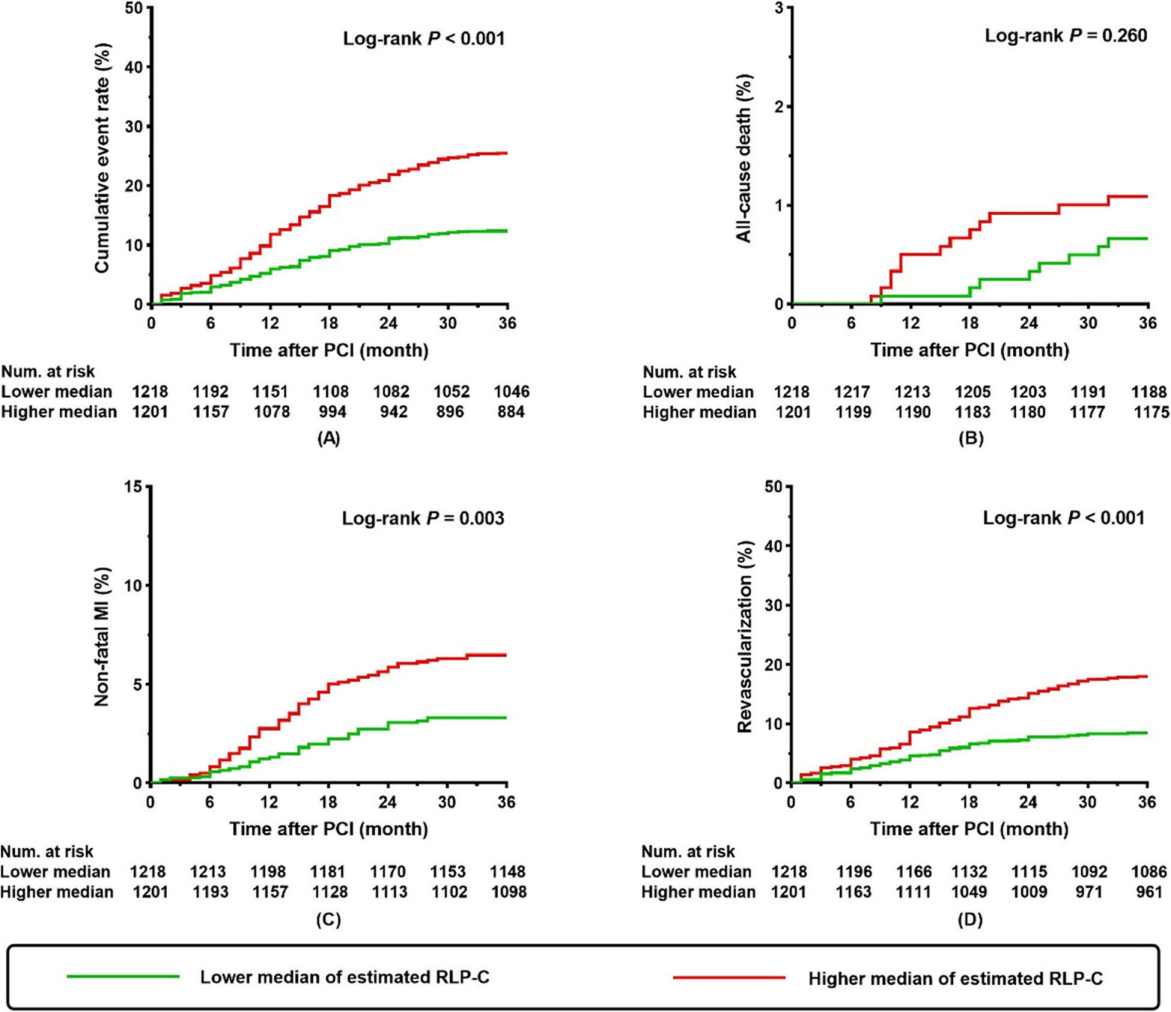
Kaplan-Meier curves for cumulative event rate according to estimated RLP-C levels in the total population. Kaplan-Meier curves for (**a**) composite adverse events; (**b**) all-cause death; (**c**) non-fatal MI; (**d**) ischemia-driven revascularization. RLP-C, remnant-like particle cholesterol; PCI, percutaneous coronary intervention; MI, myocardial infarction

During the follow-up period, 454 (18.8%) patients experienced an adverse event, which comprised of 21 (0.9%) all-cause deaths, 117 (4.8%) non-fatal MI, and 316 (13.1%) of ischemia-driven revascularization. Kaplan-Meier curves for the incidence of the composite and each component of adverse events were displayed in Fig. 2. Compared with a lower median of estimated RLP-C, patients with a higher median presented with a higher incidence of composite adverse events (*P* <  0.001), non-fatal MI (*P* = 0.003), and ischemia-driven revascularization (*P* <  0.001) (Fig. 2). However, there was no difference on incidence of all-cause death between groups (*P* = 0.260) (Fig. 2).

Multiple Cox analysis adjusted for variables that were significant (*P* <  0.05) in simple Cox analysis (shown in Suppl. materials: Table S1) were constructed to evaluate the impact of estimated RLP-C on predicting composite and each component of the adverse events. After adjustment of the confounding factors, increased estimated RLP-C levels were consistently observed to be a significant predictor for adverse prognosis, despite regarding estimated RLP-C as a continuous or nominal variable (Table 2).

**Table 2:** Multiple Cox analysis on predictive value of estimated RLP-C for composite and each component of adverse events in the total population

	As a nominal variable^a^				As a continuous variable^b^			
	β	HR	95% CI	*P*	β	HR	95% CI	*P*
Composite adverse events	0.673	1.960	1.558–2.465	**< 0.001**	0.256	1.291	1.119–1.490	**< 0.001**
All-cause death	0.792	2.207	0.612–7.959	0.226	0.604	1.829	0.837–3.995	0.130
Non-fatal MI	0.633	1.883	1.195–2.966	**0.006**	0.285	1.330	1.002–1.764	**0.048**
Ischemia-driven revascularization	0.608	1.836	1.395–2.416	**< 0.001**	0.189	1.208	1.016–1.438	**0.033**

Bold values indicate statistically significant associations

Multiple Cox analysis was adjusted for confounders that are significant (*P* <  0.05) in simple Cox analysis (details shown in Suppl. materials: Table S1)

*HR* Hazard ratio, *CI* Confidence interval, *MI* Myocardial infarction

^a^ The HR was examined regarding the lower median of estimated RLP-C as reference

^b^ The HR was examined by per 1-SD increase of estimated RLP-C

**Fig. 3:**
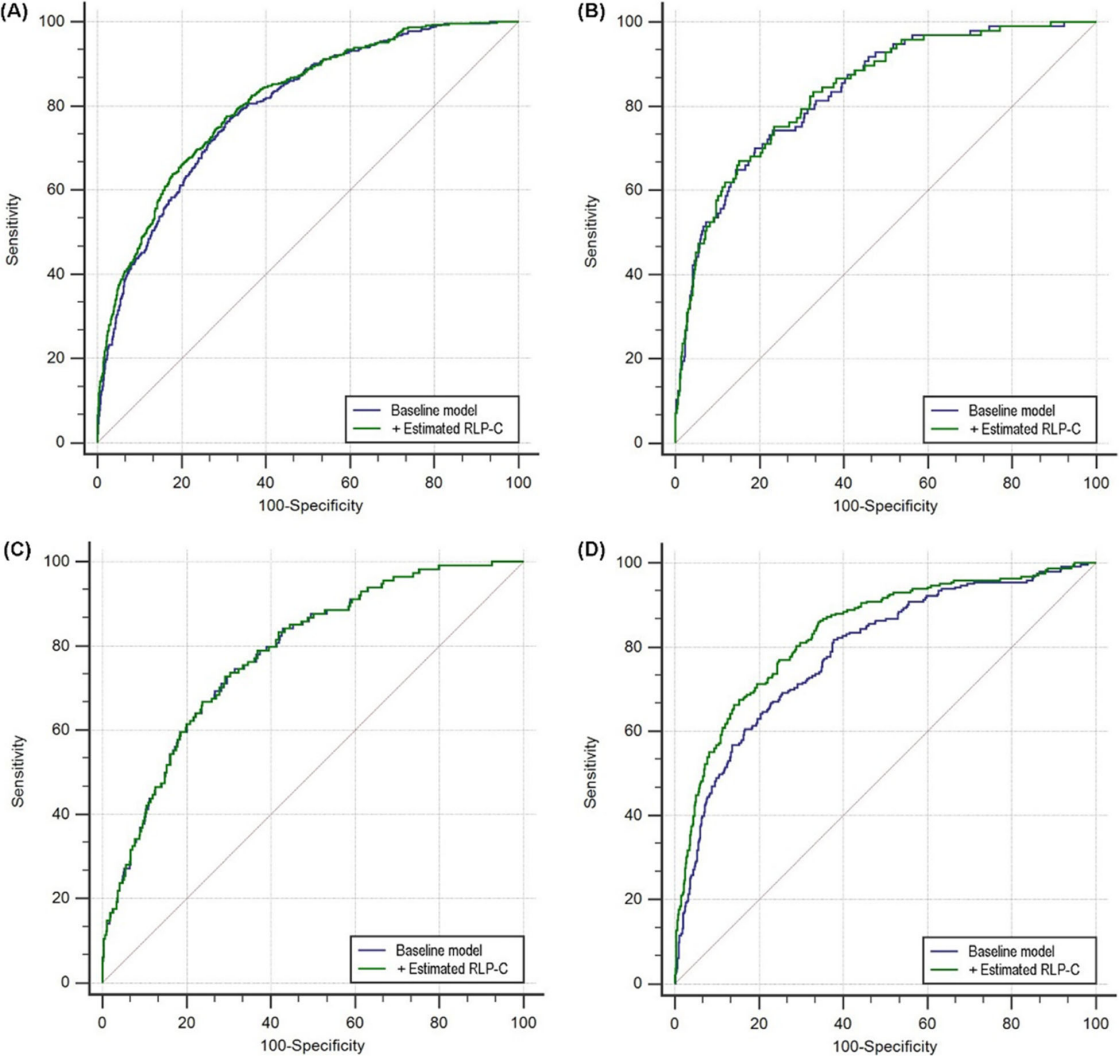
ROC curve evaluating the predictive value of various models for composite adverse events in total population and subgroups. **a** Total population; **b** Non-diabetic population; **c** Pre-diabetic population; **d** Diabetic population. The baseline model includes traditional risk factors: age, sex (female), smoking, hypertension, prior MI, prior PCI, eGFR, HbA1c, TC, HDL-C, LVEF, left main disease and multi-vessel disease. RLP-C, remnant-like particle cholesterol

The addition of estimated RLP-C enhanced the AUC obtained from the baseline model adjusted for traditional risk factors including age, sex (female), smoking, hypertension, prior MI, prior PCI, eGFR, HbA1c, TC, HDL-C, LVEF, left main disease, and multi-vessel disease (0.798 for baseline model vs. 0.811 for baseline model + estimated RLP-C, *P* for comparison < 0.001) (Table 3, Fig. 3a). Moreover, adding estimated RLP-C to the baseline model improved the discriminative performance for prediction of adverse events (category-free NRI 0.084, *P* = 0.048; IDI 0.017, *P* = 0.030) (Table 3).

**Table 3:** C-statistics for discrimination ability of the various predictive model for composite adverse events in the total population

	ROC curve analysis			Category-free NRI		IDI	
	AUC	95% CI	*P*	index	*P*	index	*P*
Baseline model^a^	0.798	0.781–0.814	reference	–	reference	–	reference
+ estimated RLP-C	0.811	0.795–0.826	**< 0.001**	0.084	**0.048**	0.017	**0.030**

Bold values indicate statistically significant associations

*ROC* Receiver operating characteristics, *AUC* Area under the curve, *CI* Confidence interval, *NRI* Net reclassification improvement, *IDI* Integrated discrimination improvement, *RLP-C* Remnant-like particle cholesterol

^a^ Baseline model includes traditional risk factors: age, sex (female), smoking, hypertension, prior MI, prior PCI, eGFR, HbA1c, TC, HDL-C, LVEF, left main disease and multi-vessel disease

### Predictive value of estimated RLP-C in subgroups with various glycometabolic status

**Fig. 4:**
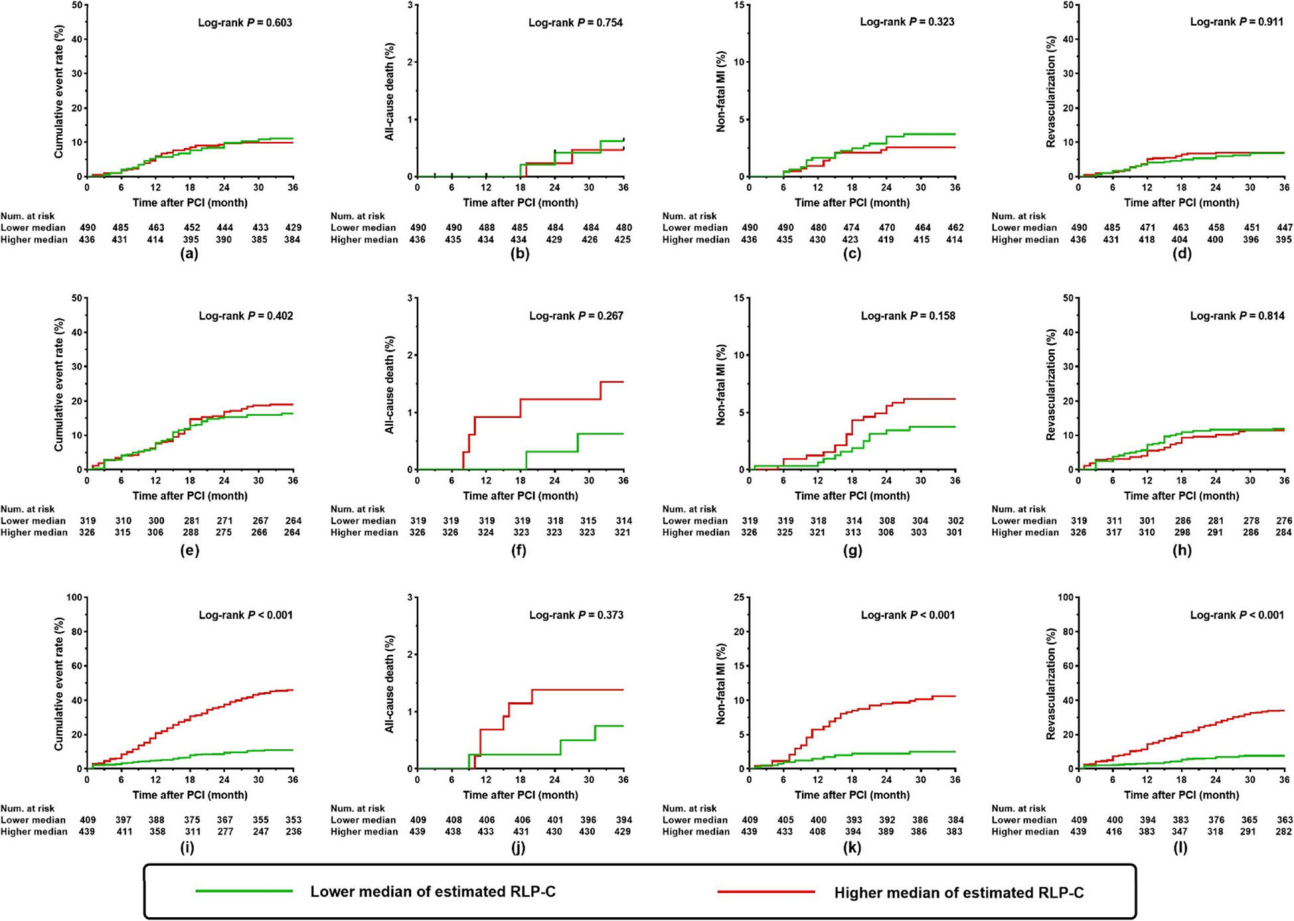
Kaplan-Meier curves for cumulative event rate according to estimated RLP-C levels in subgroups with different glycometabolic status. Kaplan-Meier curves for cumulative event rate in **a**-**d** non-diabetic population; **e**-**h** pre-diabetic population; **i**-**l** diabetic population. RLP-C, remnant-like particle cholesterol; PCI, percutaneous coronary intervention; MI, myocardial infarction

The predictive performance of estimated RLP-C was further evaluated in subgroups with various glycometabolic status [non-diabetic population (*n* = 926), pre-diabetic population (*n* = 645), diabetic population (*n* = 848)]. Kaplan-Meier curves for the cumulative rate of the composite and each component of adverse events in various subgroups were summarized in Fig. 4. In patients with diabetes, those with a higher median of estimated RLP-C, as opposed to a lower median, exhibited a higher cumulative rate of composite adverse events, non-fatal MI, and ischemia-driven revascularization, (all *P* <  0.001) (Fig. 4i-l). The difference was not found in pre-diabetic (Fig. 4e-h) and non-diabetic (Fig. 4a-d) patients.

In multiple Cox analysis, higher level of estimated RLP-C was shown to be prominently correlated to an increasing risk of adverse events in the diabetic population. However, estimated RLP-C failed to be a significant determinant of adverse events in the pre-diabetic and non-diabetic populations (Table 4).

**Table 4:** Multiple Cox analysis on predictive value of estimated RLP-C for composite and each component of adverse event in subgroups with different glycometabolic status

	As a nominal variable^a^				As a continuous variable^b^			
	β	HR	95% CI	*P*	β	HR	95% CI	*P*
Non-diabetic population								
Composite adverse events	0.177	1.193	0.681–2.092	0.538	−0.044	0.957	0.548–1.670	0.876
All-cause death	−1.067	0.344	0.001–229.549	0.748	1.421	4.143	0.240–71.536	0.328
Non-fatal MI	0.173	1.189	0.382–3.703	0.766	0.088	1.092	0.309–3.855	0.892
Ischemia-driven revascularization	0.256	1.292	0.664–2.513	0.451	−0.208	0.812	0.421–1.568	0.535
Pre-diabetic population								
Composite adverse events	0.289	1.335	0.852–2.092	0.208	−0.107	0.898	0.577–1.397	0.633
All-cause death	1.058	2.882	0.337–24.651	0.334	0.124	1.132	0.305–4.202	0.853
Non-fatal MI	0.297	1.346	0.532–3.404	0.530	0.141	1.152	0.535–2.483	0.718
Ischemia-driven revascularization	0.271	1.312	0.750–2.293	0.341	−0.321	0.725	0.405–1.297	0.278
Diabetic population								
Composite adverse events	1.446	4.247	2.941–6.135	**< 0.001**	0.326	1.385	1.183–1.620	**< 0.001**
All-cause death	0.452	1.571	0.247–9.996	0.632	−0.284	0.753	0.329–1.723	0.502
Non-fatal MI	1.804	6.072	2.669–13.815	**< 0.001**	0.331	1.392	0.975–1.988	0.069
Ischemia-driven revascularization	1.304	3.683	2.397–5.657	**< 0.001**	0.283	1.327	1.100–1.600	**0.003**

Bold values indicate statistically significant associations

Multiple Cox analysis was adjusted for confounders that are significant (*P* <  0.05) in simple Cox analysis (details shown in Suppl. materials: Table S1)

*HR* Hazard ratio, *CI* Confidence interval, *MI* Myocardial infarction

^a^ The HR was examined regarding the lower median of estimated RLP-C as reference

^b^ The HR was examined by per 1-SD increase of estimated RLP-C

The increased AUC resulting from adding estimated RLP-C to the baseline model was significant in the diabetic population (0.788 for baseline model vs. 0.836 for baseline model + estimated RLP-C, *P* for comparison < 0.001) (Table 5, Fig. 3d). By contrast, the incremental effect on AUC was not seen in the pre-diabetic and non-diabetic populations (Table 5, Fig. 3b and c). Furthermore, adding estimated RLP-C to the baseline model had a great improvement on the ability of predicting adverse events in the diabetic population (category-free NRI 0.155, *P* = 0.010; IDI 0.040, *P* <  0.001), but the additional effect was not found in the pre-diabetic and non-diabetic populations (Table 5).

**Table 5:** C-statistics for discrimination ability of the various predictive model for composite adverse events in subgroups with different glycometabolic status

	ROC curve analysis			Category-free NRI		IDI	
	AUC	95% CI	*P*	index	*P*	index	*P*
Non-diabetic population							
Baseline model^a^	0.836	0.810–0.859	reference	–	reference	–	reference
+ estimated RLP-C	0.838	0.813–0.861	0.311	0.022	0.517	0.002	0.169
Pre-diabetic population							
Baseline model^a^	0.781	0.747–0.812	reference	–	reference	–	reference
+ estimated RLP-C	0.781	0.747–0.812	0.581	0.017	0.842	0.001	0.642
Diabetic population							
Baseline model^a^	0.788	0.759–0.815	reference	–	reference	–	reference
+ estimated RLP-C	0.836	0.809–0.860	**< 0.001**	0.155	**0.010**	0.040	**< 0.001**

Bold values indicate statistically significant associations

*ROC* Receiver operating characteristics, *AUC* Area under the curve, *CI* Confidence interval, *NRI* Net Reclassification improvement, *IDI* Integrated discrimination improvement, *RLP-C* Remnant-like particle cholesterol

^a^Baseline model includes traditional risk factors: age, sex (female), smoking, hypertension, prior MI, prior PCI, eGFR, HbA1c, TC, HDL-C, LVEF, left main disease and multi-vessel disease

## Discussion

The current study confirmed an independent relationship between estimated RLP-C and recurrent adverse events in patients with NSTE-ACS undergoing PCI. Further subgroup analyses elucidated that estimated RLP-C showed a better predictive value in the diabetic population. However, estimated RLP-C failed to be an important determinant of worse outcomes in the pre-diabetic and non-diabetic populations. Adding estimated RLP-C to traditional risk factors exhibited a significant enhancement on the performance of predicting adverse events.

It has been widely demonstrated that LDL-C is one of the most significant risk indicators for ASCVD, and reduction of serum LDL-C levels with statins is a well-established therapy to reduce the ASCVD risk. However, many patients whose LDL-C levels are well controlled by statins continue to suffer recurrent cardiovascular events [3–7]. In recent years, factors related to obesity and metabolic syndrome, such as triglycerides rich lipoproteins (TRLs), have been considered as potential metabolism-related risk factors for cardiovascular diseases and possible cause of residual risks other than LDL-C. As the cholesterol component of the subset of TRLs, RLP-C has been demonstrated to be a causal risk factor of coronary artery disease (CAD) [23–25]. Previous studies also revealed that higher RLP-C levels showed favorable predictive value for adverse prognosis, either in those with stable CAD or ACS, regardless of the baseline treatment of statins and level of LDL-C [12, 26–29].

The relationship between RLP-C and plaque characteristics of coronary arteries including plaque burden, composition, and vulnerability has been disclosed by certain studies. Lina et al. revealed that RLP-C were significantly associated with plaque burden evaluated by coronary computed tomography angiography (CCTA), independent of the baseline levels of LDL-C [30]. Study from Puri et al. showed that non-HDL-C levels, which is highly correlated with RLP-C, were closely associated with the progression and regression of atherosclerotic plaque burden assessed by intravascular ultrasound, regardless of LDL-C levels [31]. Findings from Matsuo et al. revealed that in statin-treated patients, RLP-C levels, as opposed to LDL-C levels, were strongly associated with plaque vulnerability evaluated by the proportion of plaque necrosis [32]. These findings provide important confirmation and interpretation of results from previous clinical studies, suggesting that RLP-C is an important indicator of coronary atherosclerosis. Additionally, this correlation between RLP-C and plaque characteristics was observed in patients with an optimal level of LDL-C, indicating that RLP-C may be a residual risk factor on the basis of statin treatment.

In this study, the LDL-C level did not show a predictive value for poor prognosis, which was consistent with previous studies [5, 13, 29]. The underlying causes can be complex. Firstly, most participants that were enrolled in the present study underwent statin therapy, whose lipid-lowering effects in conjunction with other effects may have potential impacts on the association of LDL-C levels with adverse events. Moreover, patients with complex coronary lesions or clinical conditions may be inclined to receive more intensive lipid-lowering therapy. Such treatment selection bias or so-called “confounding by indication” may have a certain influence on the predictive ability of LDL-C. Additionally this may lead to a paradox phenomenon, such as the phenomenon present in current study that the use of ACEI could predict adverse events. The present study revealed that the estimated RLP-C remained a predictor of adverse prognosis despite the probable influence of statin treatment, which indicates that the estimated RLP-C may have greater atherogenicity than other serum lipid parameters. TGs, TC, and HDL-C lost their predictive value in multiple Cox analysis, which may partly be attributed to the strong correlation between them and the estimated RLP-C.

Results from former studies have revealed that the impact of RLP-C seems to be more prominent in patients with metabolic syndromes or type 2 diabetes [12–16]. The current study also revealed that the predictive value of estimated RLP-C is significant only in patients with diabetes, which indicates that there is significant interaction between glycometabolic status and estimated RLP-C on risk prediction. Diabetic patients have more complex lipid metabolism disorders than non-diabetic patients characterized by increased TGs and decreased HDL-C [33]. Therefore, lipid-metabolic indicators except for LDL-C may also have certain impacts on the cardiovascular risk of diabetic patients. Previous studies have proven that hypertriglyceridemia and high TRLs are important predictors for CAD [2, 4, 9]. As the major carrier of TGs, TRLs binds to the arterial endothelium, where TGs are hydrolyzed by lipoprotein lipase, finally resulting in the generation of remnant lipoproteins. Thus, the level of TGs is closely related to the cholesterol component of remnant lipoproteins, namely, the RLP-C [34, 35]. The significant association of estimated RLP-C with TGs was also verified in the present study. Studies have also showed that RLP-C increased in patients with diabetes compared with those without [12, 26, 35], which was consistent with the present study. These may all contribute to the significant prognostic impacts of RLP-C in patients with recognized diabetes.

Several pathophysiologic mechanisms may account for the relationship between RLP-C and the risk of recurrent adverse events. These include: (1) RLP-C can upregulate the expression of proinflammatory cytokines, which facilitate the monocytes moving into the arterial wall [36]; (2) RLP-C increases the generation of tissue factors (TF), which is essential for the formation of thrombus in vessels [36]; (3) There is evidence that RLP-C can enhance the aggregation of platelets [37]; (4) RLP-C promotes the propagation of smooth muscle cells that is independent from the impact of oxidative stress [38]; (5) RLP-C is causally related to low-grade inflammation, with a nearly triple increase in CRP for every 1 mmol/L increase in RLP-C [39]; (6) RLP-C was demonstrated to be an indicator of endothelial vasomotor dysfunction [16, 40]; (7) High concentrations of RLP-C were proven to be correlated to inflammation in the arterial wall in cases of intimal damage [41]. The pro-inflammatory and pro-atherothrombotic roles of RLP-C listed above may be the explanation for the association between RLP-C and cardiovascular disease.

Studies have shown that less than a quarter of patients exhibited an LDL-C level below the guideline-recommended target [28, 42]. This discordance between target value and clinical practice is often called “treatment gap”, which is a prevalent problem in the real world. In this context, while regarding LDL-C as the major target, the exploration of residual risk factors, such as RLP-C, can also provide complementary therapeutic strategies for reducing cardiovascular risk. Previous reports showed that lipid-lowering agents, such as fibrates, ezetimibe, and statins, as well as diet adaptation, proper aerobic exercise, and obesity reduction, may effectively decrease RLP-C levels to varying degrees [26, 43, 44], thus enabling RLP-C as a therapeutic target. Clinical trials of non-statin, lipid-lowering treatments have shown significant benefit in reducing residual risk, but none have specifically targeted RLP-C. Studies have demonstrated that omega-3 fatty acid derivatives [45] and antisense oligonucleotide to apolipoprotein C-III [46] have the potential to reduce TRLs significantly and provide useful tools for answering this question. In the JELIS study, eicosapentaenoic acid (an omega-3 fatty acid derivative) combined with low-dose statins reduced triglycerides by about 5% and coronary events by 19% compared to low-dose statins alone [47]. Furthermore, novel agents like inhibitors of apolipoprotein C-III and antibodies to PCSK9 were also proven to have promising results [48, 49]. Nowadays, the pattern of targeting LDL-C alone has changed, with recent guidelines highlighting the important role of non-HDL-C, which includes RLP-C, on the pathogenesis of atherosclerosis and thus its availability as an additional therapeutic target [11]. It is necessary to develop new therapies targeting RLP-C and conduct randomized trials evaluating whether lowering RLP-C levels can regulate plaque morphology and reduce the residual risk for cardiovascular outcomes.

### Study strengths and limitations

This observational cohort study expanded the relationship between estimated RLP-C and poor outcomes to a population diagnosed with NSTE-ACS and received PCI treatment. The major strengths of the present study were the large number of enrolled subjects and the long follow-up period. Additionally, the prognostic impact of estimated RLP-C was evaluated in patients with differing glycometabolic status. There are some limitations in the present study: (1) In the fasting state, VLDL remnants are the primary constituent of circulating remnants, so the contribution of chylomicron remnants may have been underestimated [50]. (2) Although potentially not as accurate as direct measurement, especially among patients with elevated TGs [51], estimated RLP-C as used in the present study is easy to calculate by using routine lipid profiles and requires no additional expense. (3) The information about the type and dosage of statins, as well as other non-statin lipid-lowering agents is relatively scarce. (4) Finally, although sequential surveillance may provide more information, only baseline lipid profiles were obtained in the current study.

## Conclusions

Estimated RLP-C is significantly associated with the recurrent adverse events in patients with diabetes and NSTE-ACS treated with PCI, as opposed to in the subgroup of pre-diabetic and non-diabetic populations. Adding estimated RLP-C to traditional risk factors significantly promotes the predictive performance for adverse events, especially in diabetic patients. The current study indicated that the evaluation of estimated RLP-C is important, not only for evaluating the risk of adverse prognosis, but also for tailoring treatment to prevent impending cardiovascular events in specific populations, such as diabetic patients. Further studies investigating whether appropriate therapeutic strategies targeting estimated RLP-C levels can improve the prognosis of CAD patients are needed to be proceeded.

## Supplementary information

**Additional file 1: Table S1.** Simple and multiple Cox analysis for composite adverse events (variates that are not significant in simple Cox analysis are not listed).

## Data Availability

The datasets generated and analyzed for this study are available from the corresponding author upon reasonable request.

## Abbreviations

ASCVD: Atherosclerotic cardiovascular disease
LDL-C: Low-density lipoprotein cholesterol
ACS: Acute coronary syndrome
RLP-C: Remnant-like particle cholesterol
non-HDL-C: Non-high-density lipoprotein cholesterol
NSTE-ACS: Non-ST-segment elevation acute coronary syndrome
PCI: Percutaneous coronary intervention
HbA1c: Glycosylated hemoglobin A1c
TGs: Triglycerides
TC: Total cholesterol
HDL-C: High-density lipoprotein cholesterol
eGFR: Estimated glomerular filtration rate
MI: Myocardial infarction
HR: Hazard ratio
CI: Confidence intervals
SD: Standard deviation
ROC: Receiver-operating characteristic
BMI: Body mass index
SBP: Systolic blood pressure
hs-CRP: High-sensitivity C-reactive protein
FBG: Fasting blood glucose
LVEF: Left ventricular ejection fraction
AUC: Area under the curve
NRI: Net reclassification improvement
IDI: Integrated discrimination improvement
TRLs: Triglycerides rich lipoproteins
CAD: Coronary artery disease
ACEI: Angiotensin-converting enzyme inhibitors
VLDL: Very-low-density lipoprotein
