# Supplementary material for: Prognostic impact of estimated remnant-like particle cholesterol in patients with differing glycometabolic status: an observational cohort study from China
Source: Lipids Health Dis. 2020 Jul 31;19:179. doi: 10.1186/s12944-020-01355-y (PMC7393817; doi:10.1186/s12944-020-01355-y)
Supplement: Supplementary file 1 — Additional file 1: Table S1. Simple and multiple Cox analysis for composite adverse events (variates that are not significant in simple Cox analysis are not listed). [file 12944_2020_1355_MOESM1_ESM.docx]

Table S1. Simple and multiple Cox analysis for composite adverse events (variates that are not significant in simple Cox analysis are not listed).

|  | Simple Cox analysis | | | Multiple Cox analysis | | |
| --- | --- | --- | --- | --- | --- | --- |
|  | HR | 95% CI | *P* | HR | 95% CI | *P* |
| Age, per 10 years | 1.351 | 1.212-1.506 | <0.001 | 1.348 | 1.189-1.528 | <0.001 |
| BMI, per 3.45 kg/m² | 1.107 | 1.018-1.203 | 0.018 |  | insignificant |  |
| Heart rate, per 10 bpm | 1.148 | 1.054-1.250 | 0.001 |  | insignificant |  |
| SBP, per 10 mmHg | 1.089 | 1.032-1.150 | 0.002 | 1.068 | 1.009-1.130 | 0.023 |
| Diabetes | 2.323 | 1.931-2.793 | <0.001 |  | insignificant |  |
| Prior MI | 2.600 | 2.153-3.139 | <0.001 | 1.683 | 1.342-2.111 | <0.001 |
| Prior PCI | 2.214 | 1.809-2.709 | <0.001 | 1.683 | 1.354-2.090 | <0.001 |
| Prior CABG | 3.897 | 2.720-5.584 | <0.001 |  | insignificant |  |
| Prior stroke | 1.651 | 1.292-2.109 | <0.001 | 1.388 | 1.077-1.789 | 0.011 |
| TGs, per 1.32 mmol/L | 1.366 | 1.297-1.439 | <0.001 |  | insignificant |  |
| TC, per 1.06 mmol/L | 1.155 | 1.059-1.260 | 0.001 |  | insignificant |  |
| HDL-C, per 0.23 mmol/L | 0.753 | 0.681-0.833 | <0.001 |  | insignificant |  |
| Estimated RLP-C, per 0.42 mmol/L | 1.369 | 1.299-1.443 | <0.001 | 1.291 | 1.119-1.490 | <0.001 |
| hs-CRP, per 6.07 mg/L | 1.113 | 1.030-1.202 | 0.007 |  | insignificant |  |
| eGFR, per 20.36 ml/(min*1.73m²) | 0.865 | 0.787-0.951 | 0.003 |  | insignificant |  |
| FBG, per 1.94 mmol/L | 1.328 | 1.260-1.399 | <0.001 |  | insignificant |  |
| HbA1c, per 1.21 % | 1.550 | 1.450-1.657 | <0.001 | 1.344 | 1.174-1.539 | <0.001 |
| LVEF, per 6.81 % | 0.693 | 0.640-0.749 | <0.001 | 0.831 | 0.757-0.912 | <0.001 |
| Diagnosis, NSTEMI | 1.437 | 1.149-1.797 | 0.001 |  | insignificant |  |
| ACEI | 1.234 | 1.017-1.497 | 0.033 | 1.264 | 1.033-1.547 | 0.023 |
| Oral hypoglycemic agents | 1.768 | 1.437-2.174 | <0.001 |  | insignificant |  |
| Insulin | 2.145 | 1.681-2.738 | <0.001 |  | insignificant |  |
| Left main disease | 2.865 | 2.112-3.887 | <0.001 | 2.649 | 1.922-3.653 | <0.001 |
| Multi-vessel disease | 4.496 | 3.333-6.064 | <0.001 | 1.847 | 1.333-2.560 | <0.001 |
| Chronic total occlusion | 3.351 | 2.748-4.086 | <0.001 | 2.646 | 2.131-3.285 | <0.001 |
| Diffuse lesion | 2.012 | 1.665-2.431 | <0.001 | 1.447 | 1.182-1.772 | <0.001 |
| Bifurcation lesion | 1.556 | 1.266-1.913 | <0.001 | 1.386 | 1.118-1.720 | 0.003 |
| Number of stents, per 1 stent | 1.237 | 1.161-1.318 | <0.001 |  | insignificant |  |

BMI, body mass index; SBP, systolic blood pressure; MI, myocardial infarction; PCI, percutaneous coronary intervention; CABG, coronary artery bypass grafting; TGs, triglycerides; TC, total cholesterol; HDL-C, high-density lipoprotein cholesterol; RLP-C, remnant-like particle cholesterol; hs-CRP, high-sensitivity C-reactive protein; eGFR, estimated glomerular filtration rate; FBG, fasting blood glucose; HbA1c, glycosylated hemoglobin A1c; LVEF, left ventricular ejection fraction; NSTEMI, non-ST-segment elevation myocardial infarction; ACEI, angiotensin-converting enzyme inhibitor.
